# Optimising electronic prescribing in hospitals: a scoping review protocol

**DOI:** 10.1136/bmjhci-2019-100117

**Published:** 2020-01-27

**Authors:** Jac Williams, David W Bates, Aziz Sheikh

**Affiliations:** 1 The University of Edinburgh Usher Institute, Edinburgh, UK; 2 Division of General Internal Medicine and Primary Care, Brigham and Women's Hospital, Boston, Massachusetts, USA

**Keywords:** health care, patient care, information systems, medical informatics, record systems

## Abstract

**Introduction:**

Electronic prescribing (ePrescribing) systems can improve the quality of prescribing decisions and substantially reduce the risk of serious medication errors in hospitals. However, realising these benefits depends on ensuring that relevant sociotechnical considerations are addressed. Optimising ePrescribing systems is essential to maximise the associated benefits and minimise the accompanying risks of these large-scale and expensive health informatics infrastructures.

**Methods:**

We will undertake a systematic scoping review of the literature to identify strategies to achieve optimisation of ePrescribing systems. We will search Medline, Embase and CINAHL for the period 1 January 2010 to 1 June 2019 and the grey literature by using Google Scholar. Independent reviewers will screen the results using predefined inclusion and exclusion criteria and will extract data for narrative and thematic synthesis.

**Discussion:**

This work will be published in a peer-reviewed journal and we will ensure that the findings are both accessible and interpretable to the public, academics, policymakers and National Health Service leaders.

## Introduction

Globally, there is considerable policy interest and substantial investment being made in moving health systems from paper-based processes to digital infrastructures to improve patient safety and improve the quality and efficiency of healthcare.^1^ The benefit of large-scale digital infrastructures is most evident in relation to electronic prescribing (also known as ‘(hospital) electronic prescribing and medicines administration’ and ‘computerized physician order entry’, henceforth referred to as ePrescribing). These systems, with their embedded medication ordering and administration systems, and computerised decision support, have the potential to both restrict and hence prevent inappropriate choices and alert prescribers to situations in which patients are at increased risk of being inadvertently harmed.^2^ They also have the potential to facilitate cost-conscious, evidence-based prescribing and enable changes in the medicines use process.^3^ Studies have however found that much of the evidence of benefit of ePrescribing systems is from the USA and came from the evaluations of ‘home-grown’ systems that have been extensively customised to the needs of local providers.^4 5^ Even in the USA, nearly all systems are now commercial. Moreover, the applicability of these data to other international contexts is unclear, particularly given that healthcare providers are overwhelmingly choosing to implement ‘off-the-shelf’ commercial software solutions in international settings.^6^

Realising the potential benefits of ePrescribing depends on optimising ePrescribing systems such that the available functionality is switched on, appropriately used, integrated with other relevant health information technology (IT) and aligned with clinical workflows. Work by our group has revealed that substantial reductions in clinically important medication errors can be achieved in ways that are likely to be cost-effective, but these are not guaranteed, with the implementation of the same ePrescribing software producing very different results in different hospitals.^7^ International studies also report variable outcomes following the implementation of ePrescribing systems, with only modest evidence of a reduction in prescribing errors in some reports and also even introduction of new prescribing errors due to unintended system consequences.^8^ The Leapfrog Group developed a tool to evaluate the safety of ePrescribing systems and showed that hospitals with longer periods since implementation did not have better scores on initial testing.^9^ However, repeated and prospective testing with the tool resulted in a consistent improvement in scores by an average of 4 percentage points per year, illustrating the benefits that can be achieved through optimisation.^9^

Health information technologies are increasingly being recognised as ‘systems of systems’, developed over time in a complex, iterative and evolving process.^10^ These systems cannot be formed instantaneously, but rather, require considerable nurturing and commitment to a life-cycle perspective. Emerging work is now beginning to focus on ensuring that these large-scale and expensive health IT infrastructures are optimised to achieve the desired clinical improvements.^11^ This led us to develop a conceptual overview of approaches that can be pursued to achieve what has been described as ‘systems optimisation’.^12^ This refers to organisational efforts to maximise the benefits and minimise the risks of using this digital infrastructure to plan and deliver care.

The benefits of the substantial investment in ePrescribing will only be realised if these systems are fully and efficiently optimised such that they support national medication safety, quality and efficiency goals. By conducting a systematic scoping review of the literature, we hope to identify the range of approaches that have been used to achieve optimisation of ePrescribing systems and assess the likely acceptability, resource implications, impact and priority of these approaches for National Health Service (NHS) hospitals. In addition, we will strive to understand how to incorporate relevant lessons in relation to systems optimisation into health systems at scale. Ultimately, this work aims to develop policy-relevant insights into how best to achieve optimisation of hospital ePrescribing systems in order to improve the safety, quality and efficiency of medicines optimisation processes.

## Methods

We will undertake a systematic scoping review of the published and grey literature. A scoping review is a technique that can be applied to evidence synthesis and is used to map the current literature, key concepts and the main sources and types of evidence in a field of interest.^13^ We have chosen a scoping review rather than a formal systematic review as our aim is to develop a comprehensive overview of this landscape, which we anticipate, will be most useful for policymaking deliberations. Furthermore, we anticipate it will be very difficult to quantify the resources spent and/or effects of individual approaches that have been pursued. Adding in the additional step of formal quality assessment of papers is therefore unlikely to represent a cost-efficient use of resources for this particular exercise. We also think it will be highly unlikely that we will be able to undertake a meta-analysis of the heterogeneous body of evidence that is likely to be uncovered.

A six-staged scoping review framework was first developed by Arksey and O’Malley (box 1),^14^ and then further refined by Levac *et al.* 15

Box 1 **The six stages of the Arksey and O’Malley^14^ methodological framework for conducting a scoping review**

- Identifying the research question.
- Identifying relevant studies.
- Study selection.
- Charting the data.
- Collating, summarising and reporting the results.
- Consultation.

In their work, Levac *et al* 15 draw on their experience of using the Arksey and O’Malley^14^ framework when conducting scoping reviews and made recommendations that seek to enhance and clarify each of the six stages of the framework. In stage 1, they emphasise the importance of linking the research question to the purpose and envisioned outcome of the work. For the second stage, they focus on addressing the balance between breadth and feasibility, and in stages 3 and 4 they highlight the importance of taking an iterative approach. Levac *et al* 15 expand on stages 5 and 6, adding further rigour to the process. We will be integrating consultative phases throughout the scoping review, reflecting the importance placed by Levac *et al* 15 on this final stage in the process. We propose to follow this updated framework as the basis for our scoping protocol as this will allow us, from an early stage, to identify and address the key considerations applicable to each phase of the work.

### Stage 1: identifying the research question

The breadth of scoping reviews can be especially useful when investigating emerging or heterogeneous fields of research. However, wide-reaching research questions can lack focus, clarity and direction. To clarify and improve this crucial initial stage, Levac *et al* 15 have suggested ‘combining a broad research question with a clearly articulated scope of enquiry’. To achieve this and establish an effective search strategy, they suggest defining the concept, the target population and the health outcomes of interest. They also advise establishing a specific purpose for the work and integrating this with the envisioned outcome early in the planning process. Accordingly, we have adapted a summary of their recommendations and the implications for this scoping review (table 1).

**Table 1:** A summary of the key considerations when defining the research question, and their implications for this scoping review^15^

Key considerations when defining the research question	Implications for our scoping review
What are the important concepts to define?	Must clearly define: ePrescribing: ‘The utilisation of electronic systems to facilitate and enhance the communication of a prescription or medicine order, aiding the choice, administration and supply of a medicine through knowledge and decision support and providing a robust audit trail for the entire medicines use process.’^19^ Optimisation: ‘The activity of enhancing system capabilities and integration of subsystem elements to the extent that all components operate at or above user expectations.’^20^
Who is the target population?	The lessons from the study should be applied to the healthcare system in the NHS. Included studies should therefore reflect this.
What are the outcomes of interest?	What are the approaches being used to achieve optimisation of ePrescribing systems, and in relation to these approaches what are the: Resource implications (ie, time/money, and so on). Likely impacts (both positive and negative). Acceptability. Identify benchmark national and international hospitals and develop a detailed appreciation of the approaches they have pursued. Identify relevant lessons in relation to systems optimisation for widespread adoption across the NHS at scale.
What is the purpose of the work?	The purpose of the study is to develop policy-relevant insights into how best to achieve ePrescribing systems optimisation in the NHS.
What is the envisioned outcome?	A description of the range of approaches used for optimisation. A description of the types of ePrescribing systems that have been deployed in the trade-offs between costs and benefits. A road map of relatively easy ‘quick-win’ optimisation strategies versus optimisation strategies that are more resource intensive and difficult to achieve. A map of countries and health systems showing where the evidence in this field is originating. Develop a list of the key investigators/opinion leaders in this field. A short summary of included literature, to be disseminated as an available resource through the ePrescribing Toolkit, http://www.eprescribingtoolkit.com/.^21^

NHS, National Health Service.

**Figure 1:**
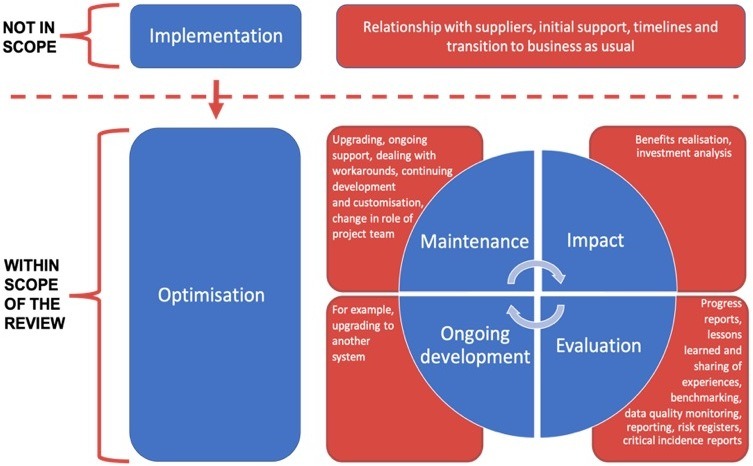
Defining the boundaries of ‘optimisation’ for the purpose of the scoping review (adapted from Cresswell *et al* [16]).

Of the key considerations proposed by Levac *et al*,^15^ we anticipate the most challenging will be to define the concepts of ‘ePrescribing’ and ‘optimisation’. While both are well-established and well-defined concepts, we acknowledge the interplay between ePrescribing systems and robotic dispensing systems deployed in pharmacies, and also recognise that the future of ePrescribing is likely to involve the use of Apps and smartphone technology. We will accordingly expand on the concept of ePrescribing to encompass this interplay with other systems and the potential for future development within the field. It is important to remember that optimisation should be conceptualised as a long journey through many stages, and the boundaries between the latter stages of implementation and optimisation are often difficult to distinguish.^12^ Following the process of implementation, ePrescribing systems become established, and enter a period referred to as a stage of maintenance.^16^ In reality, however, the maintenance of ePrescribing systems should be a dynamic process as the system adapts to evolving challenges and is subjected to regular evaluation, integrated with continuous cycles of improvement. We anticipate that defining the boundaries between implementation, optimisation and maintenance will be a challenging aspect of the work. Our scoping review will therefore focus on the strategies adopted beyond the phase of implementation to improve established ePrescribing systems (figure 1).

### Stage 2: identifying relevant studies

The next stage of the work will involve developing a robust search strategy to identify the relevant studies (online supplementary appendix 1). We will search Medline, Embase, CINAHL and Google Scholar for the period 1 January 2010 to 1 June 2019. Levac *et al* 15 suggest that a scoping review should be both broad and comprehensive, however they also recommend taking feasibility into consideration. 2010 has been chosen as the start date because this is the time from the end date of our previous evidence synthesis on health IT undertaken for NHS Connecting for Health’s Evaluation Programme.^2^ On pragmatic grounds, we will focus on human studies published in English. The Google Scholar search engine will be used to search for grey literature with the first 100 results being considered for each phrase/term searched. We will augment these searches of the published literature by liaising with an international panel of experts.

10.1136/bmjhci-2019-100117.supp1 Supplementary data

### Stage 3: study selection

Following the initial deduplication process, we will follow a systematic approach to conduct the screening phase. Based on the titles and abstracts of studies generated from our search, independent reviewers will select the relevant studies based on predefined inclusion and exclusion criteria for further full-text analysis (box 2). Disagreements will be resolved through arbitration by an independent reviewer.

Box 2 **Inclusion and exclusion criteria**

**Inclusion criteria**

- Primary studies or systematic reviews with a clearly defined methodology that describe an approach/approaches to the optimisation of an ePrescribing system.
- The study should be set in a high-income country, as defined by the Organisation for Economic Co-operation and Development (OECD).^22^

**Exclusion criteria**

- Study does not address the optimisation of an ePrescribing system.
- The study is an opinion piece or a review without a clearly defined methodology.
- Study takes place in a healthcare context that is not applicable to learning for UK National Health Service (NHS) hospitals.
- The country of the study is not within the OECD.^22^

At this stage, Levac *et al* 15 advocate taking an iterative approach by further refining the search strategy based on the abstracts retrieved. They also strongly recommend reviewing full articles for study inclusion. We propose to follow this advice by reviewing titles, abstracts and full papers before the screening phase to ensure our search strategy is identifying relevant papers. By consulting an international panel of experts in the field of ePrescribing we will already have a small number of key papers identified for potential inclusion in the scoping review. Checking that our search can identify the key papers picked by our expert panel will be another way of assessing the strength of the search strategy while also allowing for further iteration.

### Stage 4: charting the data

To chart the data, we will use a customised data extraction form when undertaking the full-text analysis of included studies (box 3).

Box 3 **Two independent reviewers will record the following data from studies**

- Title.
- Type of document.
- Lead author(s)/key investigator(s).
- Date of publication/accessed from the web.
- Source.
- Country where the study was conducted.
- Healthcare context/system (eg, private/government, specialty based/hospital wide).
- Purpose/objective of study.
- Type and version of ePrescribing system being used.
- Method/intervention used for systems optimisation.
- How was systems optimisation measured?
- Did the method/intervention used for systems optimisation result in a measurable improvement?
- Resource implications (eg, time/money).
- Impact of optimisation (positive and negative effects).
- Barriers and facilitators to optimisation that are identified.
- Lessons for optimisation.
- Summary.

As mentioned at the previous stage, Levac *et al* 15 once again advocate an iterative approach to data charting. They suggest that team members should meet initially and collectively develop the data extraction form aimed at answering the research question. However, they acknowledge that as the researchers become more familiar with the data, meeting again and refining the data extraction form will often be necessary. In fact, in their paper, Levac *et al* 15 recommend that the two researchers independently extract data from the first 5–10 studies using the data extraction form and then meet to determine whether the resulting data are sufficient for the research question and purpose. For this reason, the initial data extraction fields detailed here will be subject to iterative amendment and may not reflect the final version.

In the context of this study, and especially given the breadth of the search strategy, we anticipate that we will encounter a large volume of heterogeneous literature. To facilitate our thematic analysis, and in the interests of feasibility, we propose working backwards from the most recently published papers using principles of data saturation as we extract meaningful lessons. Saturation is a methodological principle from the field of qualitative research and is mainly used as a criterion for discontinuing data analysis or collection.^17^ While saturation as a concept continues to evolve,^17^ we identify with the 2016 definition by Given, who considered saturation as the point at which ‘additional data do not lead to any new emergent themes’.^18^ This approach of inductive thematic saturation^17^ will allow the scoping review to succinctly map the emerging themes in this diverse field of literature.

### Stage 5: collating, summarising and reporting results

Levac *et al* 15 add further rigour to the framework initially set out by Arksey and O’Malley^14^ by dividing this phase into three meaningful steps:

1. Analysing the data (this should include a descriptive numerical summary and a thematic analysis).
2. Reporting the results.
3. Applying meaning to the results.

Reflecting the three points above, our results will be analysed descriptively and thematically focusing on the following aspects:

- The range of approaches that have been applied to the optimisation of ePrescribing systems in various health systems and hospitals, drawing attention to benchmark national and international hospitals.
- A high-level indicative assessment of the resource implications and perceived impact of these approaches, both positive and negative.
- We hope to synthesise the above data to develop policy-relevant insights into how best to achieve optimisation of NHS hospital ePrescribing systems in order to improve safety, quality and efficiency of medicines management.

Given the heterogeneous nature of the studies that we anticipate encountering, quantitative analysis of the impact of different optimisation strategies is unlikely to be feasible.

### Stage 6: consultation

We propose integrating consultative phases twice during the scoping review. Having identified patient and public representatives and an international panel of experts in the field of ePrescribing, we will invite them to join our research team for meetings to help shape the development of the project and to share ideas. During the first consultation their insights will help guide the scope of the study and in the early stages will also allow us to identify areas of the grey literature to consider for inclusion. Sharing our preliminary findings from stage 5 with the panel of experts and patient and public representatives will help us identify any overlooked or outdated areas within the literature. Their involvement at this later stage will also help us refine our thematic analysis, and their input when extrapolating applicable lessons for policymakers, hospitals, healthcare workers and patients will be invaluable.

## Discussion

This scoping review will be completed as the first phase of a wider study on the optimisation of ePrescribing systems commissioned by the Department of Health and Social Care (DHSC). Our approach to this wider phased programme of work will involve detailed case studies of leading UK and international hospitals to identify potentially transferable/scalable lessons for the NHS. A series of expert round-table events, throughout the life course of the project, will help identify strategies as well as policy barriers and enablers for systems optimisation of ePrescribing software across NHS hospitals.

By following the six stages of the Arksey and O’Malley^14^ methodological framework for conducting a scoping study, and with particular attention to the additional detail and refinement added by Levac *et al*,^15^ we hope that this scoping study will lay a broad yet considered foundation for the research phases that will follow. This work will lead to a more comprehensive and nuanced appreciation of how hospitals can maximise the benefits from ePrescribing systems in order to reduce the risk of iatrogenic medication-associated harm for patients, improve the quality and efficiency of prescribing decisions and maximise return on investments. We will work closely with colleagues in the DHSC to ensure that the findings are both accessible and interpretable to policymakers and NHS leaders. We also hope to draw on the collaborative nature of this work and develop effective dissemination strategies with our expert panel and patient and public representatives. By working with stakeholders and leaders in the field we hope to share lessons, spark discussions and generate future collaboration with fellow academics. Patient and public representatives will take the lead in producing lay summaries for our research output, adding further value and impact to our scoping study.
