# Supplementary material for: Optimising electronic prescribing in hospitals: a scoping review protocol
Source: BMJ Health Care Inform. 2020 Jan 27;27(1):e100117. doi: 10.1136/bmjhci-2019-100117 (PMC7062357; doi:10.1136/bmjhci-2019-100117)
Supplement: Supplementary data [file bmjhci-2019-100117supp001.pdf]

Appendix 1 – Search Strategy

1. exp Medication Systems/ or Drug Information services/ or adverse drug reaction reporting systems/ or clinical pharmacy information systems/ or Technology, Pharmaceutical/ or Pharmaceutical Services, Online/ or Clinical Pharmacy Information Systems/ or drug therapy, computer-assisted/ or Medical Order Entry Systems/ or Electronic Prescribing/ or Decision support systems, clinical/ or Decision support techniques/ or Decision making, computer assisted/

2. (E-prescri* or Eprescri* or Electronic prescri* or "Electronic Transmission ADJ2 Prescription*" or "Computer* Physician Order Entry" or CPOE or EMAR or "electronic medication administration record" or "electronic medicines administration record" or "Hospital electronic prescribing ADJ2 medication administration" or "Hospital electronic prescribing ADJ2 medicines administration" or HEPMA or "Medic* Order Entry Systems" or ADR or "adverse drug reaction* report* system*" or "Medication system*" or "Medicine* system*" or "Medicine* administration*" or "Medication* administration*" or "Clinical decision* support" or CDSS or "decision support technique*" or "medic* management solution*").tw.

3. (Drug Prescriptions/ or Medication therapy management/) and (cell phone/ or Smartphone/ or Mobile applications/)

4. ((ePrescribing or e-prescribing or prescribing) adj3 (app* or mobile* or Smartphone*)).tw.

5. (eprescribing or e-prescribing or prescri*).tw. and (Software/ or software.tw.)

6. (Robot* and (dispens* or pharmac*)).tw.

7. ("Integrated electronic prescrib*" or "Automat* dispens*" or "Robotic prescri* dispensar*" or "Closed loop prescri*").tw.

8. 1 or 2 or 3 or 4 or 5 or 6 or 7

9. (Optimi?ation or Optima* or "System optimi?ation").tw.

10. "Quality ADJ2 healthcare"/ or Quality improvement/ or Safety/ or Patient safety/ or Efficiency/ or Clinical audit/ or Data reuse/ or cost benefit analysis/

11. (Quality or Improv* or Efficiency or audit or "Data reuse" or "System iteration" or Workaround* or "Continuous cycle* ADJ2 improvement" or "Reporting system*" or "Data quality monitoring" or "Critical incidence reports" or "End-user feedback" or Upgrad* or "Benefits reali?ation" or "Investment analysis").tw.

12. 9 or 10 or 11

13. 8 and 12

14. Limit 13 to english language

15. Limit 14 to yr="2010 –current"

16. Limit 15 to humans
